# Associations of care continuity and care coordination with the overuse of healthcare services: a nationwide population-based study

**DOI:** 10.1186/s12913-024-12099-1

**Published:** 2024-12-18

**Authors:** Guann-Ming Chang, Hsien-Yen Chang, Wen-Yu Kuo, Yu-Chi Tung

**Affiliations:** 1 Department of Family Medicine, Chang Gung Memorial Hospital, Linkou, Taoyuan, Taiwan; 2 Department of Health Policy and Management, Johns Hopkins University Bloomberg School of Public Health, Baltimore, MD USA; 3 Institute of Health Policy and Management, College of Public Health, National Taiwan University, Room 634, No.17, Xu-Zhou Road, Taipei, 100 Taiwan

**Keywords:** Continuity, Coordination, Overuse, Low-value care

## Abstract

**Background:**

Care continuity and care coordination have received increased attention as important ways of decreasing overuse/low-value care. Prior research has verified an association between care continuity and overuse or an association between care coordination and overuse. However, little is known about the relative influences of care continuity and care coordination on overuse. We used nationwide population-based data from Taiwan to examine the relative associations of care continuity and care coordination with overuse.

**Methods:**

We analyzed 1,462,960 beneficiaries in 2015 randomly sampled from all people enrolled in the Taiwan National Health Insurance. Having adjusted for patient characteristics, the multivariable logistic regression model was used to examine the associations of the Continuity of Care (COC) Index and care density on overuse, using a previously validated set of 18 potentially low-value care services.

**Results:**

Higher COC index was associated with lower overuse (low vs. medium: odds ratio [OR], 1.11; 95% confidence interval [CI], 1.09–1.12; high vs. medium: OR, 0.80; 95% CI, 0.795–0.813). Higher care density was associated with lower overuse (low vs. medium: OR, 1.01; 95% CI, 1.001–1.024; high vs. medium: OR, 0.88; 95% CI, 0.87–0.89).

**Conclusions:**

Increased care continuity and care coordination are associated with decreased overuse. Facilitating care continuity and care coordination may be an important strategy for reducing overuse/low-value care.

## Introduction

To control the rapidly rising healthcare spending that is occurring across the world, there is global interest in optimizing healthcare delivery by increasing underuse/high-value care and decreasing overuse/low-value care [1–3]. Overuse is defined as the provision of procedures and treatments that provide little or no benefit to beneficiaries while increasing the costs of healthcare [1, 4–9]. Finding the determinants that influence overuse is essential for developing effective initiatives to reduce overuse. To our knowledge, regarding health system factors, one study has identified an association between care continuity and overuse measured by the use of any 19 overuse indicators, using the United States (U.S.) Medicare data [10] and another study has documented an association between care coordination and overuse of benzodiazepines using commercial healthcare claims data [11]. However, little is known about the relative associations of care continuity and care coordination with overuse using nationwide population-based data.

In recent years, overuse has received increasing global interest in addition to underuse [3, 7, 12, 13]. Overuse has been recognized as a major contributor to the high costs of healthcare [3, 7, 13]. Using a portfolio of multiple overuse indicators as a proxy for the measurement of overuse is more appropriate than focusing on individual overuse indicators to encourage health systems to identify its system-wide determinants and thereby tackle this problem [6, 10]. Previous studies have validated that overuse, measured by multiple overuse indicators, is associated with increased medical costs and not with total mortality in the U.S. [6, 8] and in Taiwan [14].

Improving care coordination has become a crucial strategy for enhancing healthcare quality and reducing costs [15]. Continuity of care is characterized by three forms: relational continuity, informational continuity, and management continuity [16]. Care coordination involves both informational and management continuity [15, 16]. Relational continuity, also referred to as interpersonal continuity, provider continuity, or care continuity, primarily involves an ongoing relationship between a physician and a patient [15–17]. Informational continuity refers to the consistent availability of patient information, such as through follow-ups, referrals, and feedback [16–18]. Management continuity is defined as the seamless delivery of care by different physicians, often through shared management plans and care protocols, ensuring that services are provided in a complementary and timely manner [16, 17].

One study has found that increased care continuity/provider continuity, as measured by the Continuity of Care (COC) Index, is associated with a reduction in overuse, as assessed by the above-mentioned validated set of multiple overuse indicators [10]. When a patient sees fewer physicians, the likelihood of experiencing provider continuity increases, fostering an ongoing doctor-patient relationship [19, 20]. Bice and Boxerman developed the COC Index as a measure of provider continuity using claims data [10, 21, 22]. Enhanced physician continuity can lead to improved physician–patient trust [23, 24], higher patient satisfaction [25, 26], and better communication [27], which may contribute to reduced overuse due to a deeper understanding of patients’ symptoms and medical histories [10].

To date, no study has previously examined the relationship between care coordination and overuse measured by multiple overuse indicators. The more patients physicians share, the greater the likelihood of experiencing care coordination due to improved information exchange among physicians within or across health services organizations [28]. Consequently, Pollack et al. developed a care coordination measure called care density, using claims data [29–31]. This measure is based on the finding that physicians who share more patients (with sharing measured by claims for a common patient) are more likely to know one another through referrals and advice-seeking [28]. To our knowledge, one study has documented an association between increased care density and reduced overuse of benzodiazepines [11].

In response, we used nationwide population-based data from Taiwan to examine the relative associations of care continuity and care coordination with overuse as assessed by the previously validated set of multiple overuse indicators [14]. We hypothesized that a higher COC index and higher care density were associated with lower overuse.

## Methods

### Data source

We conducted a population-based cohort study using Taiwan’s national database provided by the Health and Welfare Data Science Center, Ministry of Health and Welfare (HWDC, MOHW), including the National Health Insurance (NHI) Research Database. The database, which is a deidentified secondary national database, contains individual files on NHI inpatient claims, NHI outpatient claims, NHI prescription drug claims, NHI beneficiaries, physicians, death certificates, and more. We merged the files by key variables.

Taiwan’s NHI is implemented for the entire population by the single-payer National Health Insurance Administration (NHIA). The NHIA offers a comprehensive package of benefits, including inpatient and outpatient care and prescription drugs. Almost all providers have contracts with the NHI program. Every beneficiary is free to visit any hospital or clinic without compulsory gatekeeping. Primary care is delivered in clinics, and specialist outpatient care is provided at hospitals’ outpatient departments. A beneficiary in Taiwan who directly sees a specialist in a hospital without a referral will pay a higher copayment than a person with a referral. Low-income households do not pay a copayment. Providers are paid predominantly on a fee-for-service basis.

### Study population

The study population comprised all NHI beneficiaries 18 years of age or older in 2015. We randomly sampled 2 million adult beneficiaries from the study population of about 19 million. The sample was representative of the adult beneficiary population in its distribution of demographic characteristics (sex, age, and insured amount). We excluded beneficiaries who made fewer than two outpatient visits in 2014, due to the inability to calculate physician continuity (*n* = 396,172) (as defined below), those who had fewer than two outpatient physicians in 2014, owing to the inability to calculate physician coordination (as described below) (*n* = 113,657), those with missing data (*N* = 27,112), and those who were ineligible for any low-value care service (*n* = 99). The final data set consisted of 1,462,960 patients.

### Measures of variables

#### Overuse of healthcare services

We defined the overuse of healthcare services according to the indicators of the Overuse Index (OI), previously called the Johns Hopkins Overuse Index. The OI was developed and validated as a composite measure of systematic overuse/low-value care using U.S. Medicare and commercial insurance claims data [6, 32] and Taiwan’s NHI claims data [14]. The 20 indicators of the OI included 13 diagnostic tests and medication, two tests used for screening, one test for monitoring, and four therapeutic care services. The clinical areas included primary care practice (four indicators), otolaryngology (three indicators), radiology (three indicators), cardiology (two indicators), and neurology, emergency practice, allergy, oncology, end-of-life care, urology, physical therapy, and surgery (one indicator each). However, we excluded serological tests for Helicobacter pylori and stress echocardiography for acute chest pain. Regarding screening tests for Helicobacter pylori, the NHIA does not reimburse serological tests. Concerning diagnostic tests for acute chest pain, the rate of use of stress echocardiography is too rare (< 0.5‰).

#### Care continuity and care coordination

Both care continuity and care coordination were measured using national outpatient claims data in the prior year. We used the Bice-Boxerman COC Index to calculate care continuity among a patient’s outpatient physicians in 2014 [10] and Pollack et al.’s care density measure to quantify care coordination among a patient’s outpatient physicians in 2014 [29–31]. The COC Index measures the concentration of visits among a patient’s outpatient physicians [6, 10], and is calculated as follows:

$$\mathrm{COC}\,\mathrm{Index}\,=\frac{(\sum_{i=1}^P n_i^2)-N}{N^2-N}$$

Where N is the total number of visits, P is the total number of physicians, and n is the number of visits to each physician.

The COC index was selected from various continuity metrics due to its capacity to capture the overall distribution of care among patients who see multiple physicians (including primary care and specialist physicians) and have multiple medical issues. This measure was well-suited to the patients who required multiple visits with different physicians due to complex conditions to examine the association between care continuity and overuse among primary care and specialist physicians [10]. If a patient sees different physicians on each visit, their COC Index is equal to 0, meaning no continuity.

Care density is computed as the average number of shared patients per pair of a patient’s outpatient physicians, representing the degree of “patient sharing” among a patient’s outpatient physicians [31]. Several studies have verified the relationship between care density to outcomes and costs of care regarding heart failure [29, 33, 34], cancer [30, 35, 36], and diabetes mellitus [31, 37] in the United States of America and Taiwan. The numerator of care density is the sum of all shared patients among each pair of a patient’s outpatient physicians, and the denominator is the total number of pairs of outpatient physicians seen by a patient. The more patients the involved physicians share, the higher physician coordination. Care density is calculated as follows:

$$\mathrm{Care}\,\mathrm{density}\,=\frac{\sum_{i=1}^mw_i}{C_2^P}=\frac{\sum_{i=1}^mw_i}{P(P-1)/2}$$

Where ${C}_{2}^{P}$ is the number of possible combinations for pairs of physicians, P is the total number of physicians, m is the total number of possible pairs of physicians, and w is the number of shared patients for each pair of physicians.

With the skewed distributions of the COCI index and care density and hypothesized nonlinear associations with overuse, the COCI index and care density were divided into tertiles, as done in previous work [29, 31, 33].

#### Covariates

The covariates included patient sex, age, low income, rural residence, comorbid conditions, number of hospitalizations, and number of outpatient visits in the prior year based on related previous empirical studies [10, 14, 33]. Covariates were constructed using 2014 data. Low income was measured as whether the patient was enrolled as a low-income beneficiary. Rural residence was measured as whether the patient’s census tract of residence was a rural area based on the definition of urbanization published by Taiwan’s National Health Research Institutes. The Charlson-Deyo Comorbidity Index (CCI) was used to measure the degree of a patient’s comorbidities. This index is the sum of weighted scores according to the presence or absence of 17 different medical conditions. A score of 0 represents that no comorbidity exists, while higher scores show a greater burden of comorbidity.

### Statistical analysis

This study used the multivariable logistic regression model—adjusting for patient sex, age, low income, rural residence, comorbidities, number of hospitalizations, and number of outpatient visits in the prior year—to examine the association of the COC Index and care density in the prior year with the receipt of any overuse event. In sensitivity analysis, we also used the Cox proportional-hazards model, which could consider the length of survival [38] because we included patients who died during the study year. The cohort entry date for everyone was the same: Jan 1, 2015; the cohort exit date was the earliest date of: 1. the first date of low-value care use, 2. date of death, or 3. Dec 31, 2015. Patients were censored if the exit date was not the first date of low-value care use. SAS 9.4 (SAS Institute, Cary, NC) was used for the analyses. All statistical testing was two-sided at a significance level of 0.05.

## Results

Table 1 presents the patients’ characteristics. On average, the 1,462,960 patients were 47.8 years old, and 55.2% were female. A total of 235,768 patients (16.1%) experienced at least one overuse event. The mean COC index was 0.23. The mean annual number of all shared patients between a pair of patient’s outpatient physicians was 390 patients. Table 2 presents the patient characteristics by care continuity and care coordination. Chi-square tests showed significant associations of the COC index and care density with overuse (*P* < 0.001). Overuse rates among low, medium, and high care continuity were 16.3%, 18.3% and 13.8%, respectively, and those among low, medium, and high care density were 16.2%, 18.1%, 14.0%, respectively.

**Table 1:** Patient characteristics

	n	%	Mean	SD
No. of patients	1,462,960	100.0	—	—
Sex				
Female	807,966	55.2	—	—
Male	654,994	44.8	—	—
Age, y	—	—	47.8	17.6
18–44	678,329	46.4	—	—
45–64	526,574	36.0	—	—
65 +	258,057	17.6	—	—
Low income				
Yes	17,430	1.2	—	—
No	1,445,530	98.8	—	—
Rural residence				
Yes	398,891	27.3	—	—
No	1,064,069	72.7	—	—
CCI	—	—	0.5	1.1
0	1,063,545	72.7	—	—
1–2	315,004	21.5	—	—
≥ 3	84,411	5.8	—	—
No. of hospitalizations in the prior year	—	—	0.2	0.6
0	1,310,645	89.6	—	—
1	112,728	7.7	—	—
≥ 2	39,587	2.7	—	—
No. of outpatient visits in the prior year	—	—	14.0	13.4
Low (< 7)	482,296	33.0	—	—
Medium (7–14)	492,042	33.6	—	—
High (> 14)	488,622	33.4	—	—
COC Index in the prior year	—	—	0.23	0.19
Low (< 0.13)	487,748	33.3	—	—
Medium (0.13–0.29)	485,200	33.2	—	—
High (> 0.29)	490,012	33.5	—	—
Care density in the prior year	—	—	389.6	702.2
Low (< 71.2)	487,190	33.3	—	—
Medium (71.2–304.0)	487,211	33.3	—	—
High (> 304.0)	488,559	33.4	—	—
Any overuse care				
Yes	235,768	16.1	—	—
No	1,227,192	83.9	—	—

*CCI* indicates Charlson-Deyo Comorbidity Index, *COC* continuity of care

**Table 2:** Patient characteristics by COC Index and Care density in the prior year

	COC Index				Care density			
	Low	Medium	High	*P* value	Low	Medium	High	*P* value
	%	%	%		%	%	%	
Sex								
Female	58.2	50.8	56.7	< 0.001	53.8	53.4	58.5	< 0.001
Male	41.8	49.2	43.3		46.2	46.6	41.5	
Age								
18–44	60.5	38.5	40.0	< 0.001	44.5	51.4	43.2	< 0.001
45–64	28.8	41.4	37.8		36.6	35.1	36.3	
65 +	10.7	20.1	22.2		18.8	13.5	20.6	
Low income								
Yes	1.1	1.3	1.2	< 0.001	1.2	1.0	1.3	< 0.001
No	98.9	98.7	98.8		98.8	99.0	98.7	
Rural residence								
Yes	26.1	28.0	27.7	< 0.001	21.2	30.9	29.7	< 0.001
No	73.9	72.0	72.3		78.8	69.1	70.3	
CCI								
0	82.4	70.3	65.4	< 0.001	69.0	81.0	68.0	< 0.001
1–2	13.5	24.6	26.5		23.2	16.6	24.8	
≥ 3	4.1	5.0	8.2		7.7	2.4	7.2	
No. of hospitalizations in the prior year								
0	90.7	91.3	86.8	< 0.001	86.6	94.9	87.3	< 0.001
1	6.9	6.6	9.7		9.4	4.3	9.5	
≥ 2	2.5	2.1	3.5		4.0	0.8	3.3	
No. of outpatient visits in the prior year								
Low	47.3	32.1	19.4	< 0.001	42.4	32.6	23.9	< 0.001
Medium	26.9	38.0	36.1		31.7	37.0	32.2	
High	25.8	29.9	44.5		25.9	30.4	43.9	
COC Index in the prior year								
Low	—	—	—	—	30.3	32.8	36.9	< 0.001
Medium	—	—	—		27.0	36.5	36.0	
High	—	—	—		42.6	30.8	27.1	
Care density in the prior year								
Low	30.3	42.4	27.1	< 0.001	—	—	—	—
Medium	36.9	26.9	36.1		—	—	—	
High	32.8	30.7	36.7		—	—	—	
Any overuse care								
Yes	16.3	18.3	13.8	< 0.001	16.2	18.1	14.0	< 0.001
No	83.7	81.7	86.2		83.8	81.9	86.0	

*CCI* indicates Charlson-Deyo Comorbidity Index, *COC* continuity of care

Table 3 presents the overuse of health care services by care continuity and care coordination. Care continuity was related to any low-value care services and 15 low-value care services (*P* < 0.05). For any and nine low-value care services, the overuse rates among high care continuity were lower than low and medium care continuity. Care coordination was associated with any low-value care services and 11 low-value services (*P* < 0.01). In the case of any and eight low-value services, the overuse rates among high care coordination were lower than low and medium care coordination.

**Table 3:** The overuse of health care services by care continuity and care coordination in the prior year

Indicator	Total Patients Eligible, No	Events per 1000 Eligible Patients	COC Index in the prior year				Care density in the prior year			
			Low (‰)	Medium (‰)	High (‰)	*P* value	Low (‰)	Medium (‰)	High (‰)	*P* value
**Diagnostic tests**										
Laryngoscopy for sinusitis	305,242	26.0	27.8	26.6	22.3	< 0.001	25.2	27.2	25.3	0.006
Nasal endoscopy for sinusitis	305,242	11.6	11.7	12.4	10.4	< 0.001	15.5	13.0	8.0	< 0.001
Electroencephalogram in syncope	5,632	158.0	169.8	160.9	141.8	0.073	151.2	157.2	170.1	0.327
MRI in mild traumatic brain injury	21,524	11.1	13.9	10.8	8.5	0.011	12.0	10.7	10.5	0.620
Sinus CT or antibiotics for rhinosinusitis	153,352	551.2	550.7	556.3	545.4	0.004	568.7	554.5	535.3	< 0.001
Unproven allergy tests	220,350	36.3	40.9	35.5	31.7	< 0.001	43.9	36.9	29.5	< 0.001
Tumor marker studies in asymptomatic breast cancer	10,855	672.0	612.3	676.3	704.2	< 0.001	668.9	671.7	683.3	0.549
Abdomen CT with and without contrast	40,887	773.8	774.1	772.3	775.5	0.805	774.5	772.8	774.4	0.929
MRI lumbar spine for low back pain	6,815	203.8	220.7	181.8	215.1	0.001	244.7	167.9	211.4	< 0.001
Advanced imaging in low-risk prostate cancer	4,088	296.0	280.9	299.7	303.8	0.454	286.6	302.9	310.3	0.416
Preoperative chest radiograph	59,447	676.3	660.1	676.2	691.5	< 0.001	699.7	663.8	659.1	< 0.001
Thorax CT with and without contrast	25,531	593.3	559.6	593.1	621.4	< 0.001	579.6	602.1	608.1	< 0.001
**Therapeutic procedures**										
Laminectomy and/or spinal fusion	1,383,174	1.0	0.9	1.2	0.8	< 0.001	1.0	1.2	0.6	< 0.001
Hysterectomy for benign disease	802,324	1.8	1.7	2.0	1.5	< 0.001	1.9	1.9	1.5	< 0.001
Traction for low back pain	234,845	216.1	240.9	225.2	179.7	< 0.001	219.8	232.5	191.5	< 0.001
More than 1 emergency department visit in last 30 days of life	14,233	166.8	175.0	176.3	153.2	0.002	162.6	175.0	159.7	0.117
**Screening tests**										
Carotid artery stenosis screening	1,437,389	10.6	8.7	13.5	9.6	< 0.001	12.4	13.2	6.1	< 0.001
**Monitoring test**										
Routine monitoring of digoxin	31,974	9.0	5.9	7.7	12.6	< 0.001	9.9	8.6	7.8	0.302
Any overuse indicator	1,462,960	161.2	163.2	182.7	137.8	< 0.001	162.0	181.2	140.3	< 0.001

*COC* indicates Continuity of Care, *CT* computed tomography, *MRI* magnetic resonance imaging *SD* standard deviation

The result of the multivariable logistic model (shown in Tables 4) demonstrated the associations of care continuity and care coordination with overuse, after adjusting for covariates. A higher COC Index and higher care density were associated with lower overuse. Patients with high COC Index had a lower odds of overuse (odds ratio [OR], 0.80; 95% confidence interval [CI], 0.795–0.813), and those with low COC Index had a higher odds of overuse (OR, 1.11; 95% CI, 1.09–1.12) compared with those with medium COC index. Patients with high care density had a lower odds of overuse (OR, 0.88; 95% CI, 0.87–0.89), and those with low care density had a higher odds of overuse (OR, 1.01; 95% CI, 1.001–1.024) relative to those with medium care density. The robustness of our primary results were evaluated by sensitivity analysis. Results were similar when we used the Cox proportional-hazards model.

**Table 4:** Multivariable logistic and Cox regression models showing the associations of care continuity and care coordination with overuse (*n* = 1,462,960)

	OR	95% CI		*P* value	HR	95% CI		*P* value
COC index in the prior year (ref: medium)								
Low	1.11	(1.09-	1.12)	< 0.001	1.10	(1.09-	1.11)	< 0.001
High	0.80	(0.795-	0.813)	< 0.001	0.82	(0.81-	0.83)	< 0.001
Care density in the prior year (ref: medium)								
Low	1.01	(1.001-	1.024)	0.030	1.01	(1.004-	1.024)	0.006
High	0.88	(0.87-	0.89)	< 0.001	0.89	(0.88-	0.90)	< 0.001
Male	1.00	(0.99-	1.01)	0.563	0.99	(0.99-	1.00)	0.113
Age (ref: 18–44)								
45–64	1.15	(1.14-	1.16)	< 0.001	1.15	(1.13-	1.16)	< 0.001
65 +	1.12	(1.11-	1.14)	< 0.001	1.11	(1.10-	1.12)	< 0.001
Low income (ref: no)	0.90	(0.87-	0.94)	< 0.001	0.91	(0.87-	0.94)	< 0.001
Rural residence (ref: no)	0.90	(0.89-	0.91)	< 0.001	0.91	(0.90-	0.92)	< 0.001
CCI (ref: 0)								
1–2	1.29	(1.28-	1.31)	< 0.001	1.26	(1.25-	1.27)	< 0.001
≥ 3	1.75	(1.72-	1.78)	< 0.001	1.64	(1.61-	1.67)	< 0.001
No. of hospitalizations in the prior year (ref: 0)								
1	1.16	(1.14-	1.18)	< 0.001	1.14	(1.12-	1.15)	< 0.001
≥ 2	1.45	(1.41-	1.48)	< 0.001	1.38	(1.36-	1.41)	< 0.001
No. of outpatient visits in the prior year (ref: < 7)								
7–14	1.39	(1.38-	1.41)	< 0.001	1.37	(1.35-	1.38)	< 0.001
> 14	2.00	(1.97-	2.02)	< 0.001	1.91	(1.88-	1.93)	< 0.001

*CCI* indicates Charlson-Deyo Comorbidity Index, *CI* confidence interval, *COC* Continuity of Care, *HR* harzard ratio, *OR* odds ratio

## Discussion

This was the first study to examine the relative associations of care continuity and care coordination with overuse measured by multiple overuse indicators using nationwide population-based data. We found that higher care continuity and higher care coordination were associated with lower overuse.

One finding that higher care continuity was related to lower overuse is consistent with Romano et al. using 5% Medicare fee-for-service claims from 2008 [10]. Romano et al. found that a higher COC Index was associated with lower overuse measured by a previously studied set of 19 overuse indicators [10, 32]. One probable explanation is that higher care continuity makes physicians accumulate knowledge of the patient’s history, values, hopes, and fears, and thus provide less low-value care than where there is less care continuity [10, 39]. Another reason is that patients with higher care continuity are more willing to trust their care physicians and therefore receive less low-value care [39, 40].

This study made one major finding regarding the relationship between higher care coordination and lower overuse. The finding is consistent with Ong et al. regarding the propensity to co-prescribe overlapping benzodiazepines [11]. Ong et al. found that increased care density was associated with decreased overuse of benzodiazepine. Although previous studies have shown that higher care density is related to decreased costs and better outcomes for different medical conditions [29, 30, 33] and reduced benzodiazepine overuse [11], no empirical study has been conducted to examine the relationship between care density and overall overuse. It is possible that patients who see physicians with higher numbers of shared patients receive better coordinated care which involves a coherent approach to patient management among physicians through the sharing of care plans and patient information rendering them less likely to use low-value care [11, 29, 31]. Barnett et al. found that physicians with higher numbers of shared patients were more likely to obtain consults from one another, to have referral relationships, or to work in the same practice [28].

Our research had certain limitations. First, in common with other studies using administrative databases to examine factors associated with overuse [10, 11, 14, 41–45], we lacked the clinical details necessary for complex clinical risk adjustment. However, we used patient age, low income, and comorbid conditions, alongside other factors important for risk adjustment. Second, the individuals who made fewer than two outpatient visits were excluded because the COC index cannot be calculated among patients with 0 or 1 outpatient visit. Thus, the data reflect individuals who made at least two outpatient visits, which may not be generalizable to those who made fewer than two. Third, there is a possibility of misclassification bias in claims data when attributing diseases to beneficiaries involved in care. However, since we do not anticipate this misclassification to vary across different levels of care continuity and care density, it is less likely to impact our main results. Fourth, it is important to note that logistic regression does not allow for quantifying the extent of overuse—only whether overuse occurred. Fifth, our study was conducted in Taiwan, whose healthcare system may have particular aspects that cannot be extrapolated elsewhere.

Our national population-based study showed the associations of care continuity and care coordination with overuse/low-value care. To reduce structural and system-wide overuse/low-value care, it is imperative to identify the determinants of overall overuse. Higher care continuity and care coordination are associated with lower overall overuse. Policies advocating accountable care organizations are expected to encourage physicians to coordinate their care and maintain continuity with their patients in order to provide value-based care. Our findings may have implications for the implementation of the abovementioned value-based payments to facilitate care coordination and care continuity and thereby reduce low-value care.

## Data Availability

Data are available upon reasonable request. The datasets used and analyzed during the current study are not publicly available but are available from the corresponding author on reasonable request with the permission of the Ministry of Health and Welfare, Taiwan.
